# Adult attachment and relationship satisfaction among young adults: the mediating role of perceived partner responsiveness

**DOI:** 10.3389/fpsyg.2026.1880927

**Published:** 2026-10-19

**Authors:** Siqi Alice Wang, Junqing Wang

**Affiliations:** School of Health, Shandong University of Traditional Chinese Medicine, Jinan, China

**Keywords:** adult attachment, college students, perceived partner responsiveness, relationship satisfaction, young adults

## Abstract

Previous research has established that insecure attachment is negatively associated with intimate relationship satisfaction. However, the psychological mechanisms underlying these associations, particularly in young adults, warrant further investigation. Guided by attachment theory, the present study investigated the mediating role of perceived partner responsiveness (PPR) in the relationship between adult attachment dimensions (avoidance and anxiety) and intimate relationship satisfaction among Chinese college students. In a cross-sectional design, 404 college students currently involved in romantic relationships completed online questionnaires assessing attachment styles, PPR, and relationship satisfaction. Attachment avoidance and anxiety were examined simultaneously to estimate their unique associations while accounting for their shared variance. The findings suggest distinct relational patterns for the two attachment dimensions among young adults: PPR accounted for only part of the association between avoidance and relationship satisfaction, whereas the unique association involving anxiety was primarily indirect through PPR. Future research could compare different developmental stages to examine whether perceived partner responsiveness is particularly salient for relationship satisfaction during young adulthood.

As social beings, humans engage in various significant social relationships, among which intimate relationships hold a prominent place. Love, as a timeless topic in human society and life, is a central experience for young adults. According to *Sex and relationships among students: summary report* (Hillman, 2021), 32% of the undergraduates in the UK are currently in a relationship, while 41% of them have had sex during university.

Erikson's theory of psychosocial development (1950) argues that individuals in early adulthood (aged 18–25) face the core conflict of cultivating intimacy vs. avoiding isolation, ultimately striving to attain the virtue of love. Although positive emotional bonds with friends and family also contribute to the development of this capacity (Xia et al., 2024, 2025), establishing an intimate relationship remains a direct and effective way to navigate this developmental challenge (Adamczyk et al., 2022). Also, extensive research has shown that healthy intimate relationships can enhance physical and psychological wellbeing in multiple ways (Miller, 2015a; Bourassa et al., 2020).

## Adult attachment

Attachment theory, proposed by Bowlby (1969), posits that humans are born with an innate tendency to seek and maintain proximity to their primary caregivers as an adaptive mechanism for survival, accompanied by a corresponding behavioral system. Through early interactions with caregivers, children form an unconscious, automatic internal working model (IWM)—a mental representation that reflects self-worth and perceived availability of others (Verschueren et al., 1996; Lopez et al., 1998). This psychological framework allows infants to develop appropriate expectations regarding the caregiver's emotional availability and responsiveness (Whitaker et al., 1999; Johnson et al., 2010). Bowlby (1969) suggested that this model comprises two core components: the model of the self and the model of others. The former refers to one's belief about whether they are worthy of care and affection from others, while the latter pertains to the expectation of whether others can be relied upon and trusted. The attachment style formed in early childhood influences interpersonal patterns and the dynamics of intimate relationships in adulthood, giving rise to what is known as adult attachment (Hazan and Shaver, 1987). Brennan et al. (1998) further identified that self-reported items reflecting different attachment styles can be categorized into two dimensions: attachment anxiety and attachment avoidance.

The characteristics of attachment avoidance are fear of intimate relationships, distrust of others, resistance to closeness, and unwillingness to express negative emotions (Ferraro and Taylor, 2021). Due to the neglect of attachment figures in expressing emotions, individuals who avoid attachment have developed a deactivation strategy toward emotions (Simpson and Overall, 2014; Leenders et al., 2019). For example, individuals with high levels of attachment avoidance are unwilling to seek help from others when solving problems and tend to choose self-digestion. Therefore, they become very independent and are likely to emotionally distance themselves from their partners when faced with stress. In relationships, they often deny the need for attachment and ignore information related to attachment (Simpson and Overall, 2014; Kuan mak et al., 2010).

The characteristics of attachment anxiety are a strong need for intimate behavior and fear of relationships, with a strong fear of being rejected by specific individuals (Mikulincer and Shaver, 2007; Chen et al., 2020). Due to the lack of stable care and support from caregivers during childhood, individuals with attachment anxiety develop hyperactivating strategies to cope with interpersonal relationships (Simpson and Overall, 2014; Leenders et al., 2019). They believe that exaggerating their needs and clearly displaying vulnerability can lead to attention and protection (Li and Chan, 2012). In relationships, individuals with high attachment anxiety tend to be very engaged and also exhibit strong fear and anxiety about separation, as they greatly need this emotional connection with others (Mikulincer and Shaver, 2007; Oon-arom et al., 2021).

## Intimate relationship and satisfaction

Relationship satisfaction, as the most common indicator for measuring the quality of intimate relationships, refers to an emotional response based on subjective evaluations of both positive and negative dimensions in romantic relationships (Fallis et al., 2016). Intimate relationship satisfaction has been shown to be an important predictor of happiness and life satisfaction in both cross-sectional and longitudinal studies (Candel and Turliuc, 2019). Previous studies have demonstrated the relationship between insecure attachment and intimate relationship satisfaction: both the deactivation strategies used by attachment avoidant individuals and the hyperactivation strategies used by attachment anxious individuals have a negative impact on intimate relationship satisfaction (Li and Chan, 2012; Vollmann et al., 2019). Individuals with attachment avoidance tend to be psychologically and behaviorally independent. When entering an intimate relationship, they do not value this sense of closeness (Guzmán-González et al., 2020), nor do they believe that their partner's care will help them. Individuals with attachment anxiety, on the other hand, have different mechanisms. They have a high emotional need for a relationship and are sensitive to signals of rejection or potential abandonment (Mikulincer and Shaver, 2007), and tend to attribute such signals negatively (Overall et al., 2015). This makes their relationship satisfaction lower. Research has found that attachment avoidance can damage the positive aspects of intimacy, while attachment anxiety tends to promote the negative aspects of intimate relationships, such as conflict (Li and Chan, 2012). However, more research is still needed in the future to further understand the specific coping strategies and psychological defense mechanisms exhibited by individuals with high avoidant attachment and high anxiety attachment in specific contexts (Mikulincer and Shaver, 2019).

## Attachment and partner responsiveness

There is likely a close connection between insecure attachment and perceived partner responsiveness. According to attachment theory (Bowlby, 1969), internal working models guide individuals “attitudes toward others” behaviors and influence their reactions to social experiences. Perceived partner responsiveness is the extent to which individuals feel that their partners, through supportive actions, understand, validate, and care for their core self (Reis et al., 2004; Holmstrom and Lim, 2023). The internal working models of individuals with insecure attachment influence their perception and interpretation of their partner's behaviors, leading them to evaluate their partner's responsiveness as lower. Individuals with attachment avoidance, due to early attachment experiences where emotional expressions were often ignored, develop “deactivation strategies” regarding intimacy needs. They tend to avoid closeness, maintain emotional independence in relationships (Simpson and Overall, 2014), and often disregard or downplay attachment-related signals (Girme et al., 2015). Consequently, even if partners demonstrate care or support in actual interactions, those with attachment avoidance may struggle to accurately recognize or attribute positive meaning to such behaviors, sometimes even perceiving them as irrelevant or intrusive (Girme et al., 2015). This active neglect and negative interpretation of relational cues result in a significantly reduced perception of partner responsiveness. In contrast, individuals with attachment anxiety, having experienced inconsistent caregiver responses in childhood, develop “hyperactivation strategies.” These manifest as high investment in relationships and intense fear of rejection. They persistently seek attention and reassurance from their partners and expect equally intense and timely responses to their emotional needs (Mikulincer and Shaver, 2007; Simpson and Overall, 2014). However, these high expectations often clash with reality. When partners fail to meet the intensity or frequency of their emotional demands, individuals with attachment anxiety tend to interpret this as rejection or indifference, leading to disappointment and distress. Even when partners do provide some level of responsiveness, their insufficient sense of security often leads them to underestimate its quality or intent (Overall et al., 2015), thereby leading them to perceive lower partner responsiveness. Although attachment avoidance and attachment anxiety differ in their manifestations, both impair the perception and evaluation of a partner's actual responsive behaviors, resulting in generally lower perceived partner responsiveness.

## The present study

Although previous research has established associations among attachment insecurity, perceived partner responsiveness, and relationship satisfaction, less is known about how these relational processes operate during young adulthood. Young adulthood is an important developmental period for establishing intimacy and developing patterns of romantic relating (Erikson, 1950; Xia et al., 2018; Suh and Fabricius, 2020). From an attachment perspective, internal working models shape expectations regarding the availability and responsiveness of close others and guide how attachment-relevant interpersonal information is interpreted (Bowlby, 1969). Perceived partner responsiveness may therefore be particularly informative in this developmental context because it captures individuals' relationship-specific perceptions of whether a current partner understands, validates, and cares for them (Reis et al., 2004). Examining PPR among young adults thus extends existing work by considering how broader attachment orientations are associated with evaluations of responsiveness within developing romantic relationships, rather than focusing only on the direct association between attachment insecurity and relationship satisfaction. Moreover, because attachment anxiety and avoidance involve different regulatory strategies, examining both dimensions simultaneously may clarify whether perceived responsiveness is similarly relevant to both forms of insecurity or whether distinct patterns emerge.

The present study sought to investigate the psychological processes linking adult attachment to intimate relationship satisfaction within a sample of Chinese college students. Guided by attachment theory (Bowlby, 1969) and Erikson's psychosocial developmental framework, which highlights young adulthood as a critical period for establishing intimacy, the present research had two primary objectives.

First, the present study aimed to examine how the two dimensions of insecure attachment, avoidance and anxiety, are associated with individuals' subjective evaluations of their relationship quality, anticipating that both would be negatively associated with relationship satisfaction at the bivariate level. Second, and more centrally, it proposed and tested a mediation model to illuminate the underlying process, hypothesizing that perceived partner responsiveness would help account for the associations between insecure attachment and relationship satisfaction. Because attachment avoidance and attachment anxiety are moderately correlated, the two dimensions were examined simultaneously, allowing the unique contribution of each to be estimated while controlling for the other.

By focusing on Chinese young adults, the present study extends existing attachment and responsiveness research to a developmental period in which establishing and consolidating intimate relationships is particularly salient.

## Method

### Participants and procedure

This study employed a mixed-mode survey strategy, distributing questionnaires via social media platforms and via in-person QR code scanning. Participants were recruited from a college student population currently involved in intimate relationships. The final sample comprised 404 valid responses, including 378 undergraduates (93.6%) and 26 graduate students (6.4%). Prior to participation, all individuals were informed that the research aimed to investigate intimate relationship satisfaction among college students. Participation involved completing a series of online questionnaires consecutively, with an estimated total duration of 25 min. As compensation, participants could choose between a cash incentive (1–5 CNY) or a set of electronic academic materials (All participants opted for the cash incentive).

### Power analysis

A Monte Carlo simulation-based power analysis was conducted in R (Version 4.5.3) to assess the sample size required to detect the hypothesized indirect associations (Schoemann et al., 2017). Using population parameters informed by previous research on attachment, perceived partner responsiveness, and relationship satisfaction (Yang et al., 2019), with α = 0.05 and a conventional target power of 0.80 (Cohen, 1988), approximately 350 participants were required to provide adequate power for both anticipated indirect associations. The final analytic sample of 404 participants exceeded this criterion.

### Measures

The online questionnaire used in this study includes questions measuring demographic variables, three scales (attachment avoidance and attachment anxiety, perceived partner responsiveness, and intimate relationship satisfaction), and two attention-check items.

#### Experiences in Close Relationships inventory (ECR)

The Experiences in Close Relationships Inventory (ECR) consists of 36 items, divided into two dimensions: attachment avoidance and attachment anxiety. The odd-numbered items measure attachment avoidance, while the even-numbered items measure attachment anxiety. This scale uses a seven-point Likert scale, ranging from 1 (“strongly disagree”) to 7 (“strongly agree”), with questions 3, 15, 19, 22, 25, 27, 29, 31, 33, and 35 reverse-scored. The questionnaire requires participants to complete it based on their current intimate relationship experience. Take the average score on each dimension as the level of insecure attachment on that dimension. The higher the score on the avoidance dimension, the greater the degree of attachment avoidance; the higher the score on the anxiety dimension, the greater the degree of attachment anxiety. When Li and Kato (2006) revised the Chinese version of ECR, the internal consistency coefficients of avoidance and anxiety dimensions were 0.82 and 0.77, respectively, indicating high reliability. This study also conducted an internal consistency test using the Alpha coefficient for the scale, yielding 0.838 and 0.908, respectively.

#### Perceived Partner Responsiveness Scale (PPRS)

The Perceived Partner Responsiveness Scale (PPRS; Reis et al., 2004) is a 12-item, unidimensional measure assessing the extent to which individuals perceive their partner as understanding, valuing, and caring for their core self. All items are positively worded and capture perceptions of responsive (rather than unresponsive) partner behavior; higher scores therefore indicate greater perceived responsiveness. Sample items include “My partner truly listens to me,” “My partner understands me,” and “My partner responds to my needs in a timely way.” Items are rated on a 7-point Likert scale (1 = “completely disagree” to 7 = “completely agree”). The Chinese version of the PPRS was revised and validated by Yang et al. (2019), who employed a double-blind translation procedure (translation and back-translation) and reported a single-factor structure with good model fit, satisfactory criterion-related validity, and strong reliability (Cronbach's α = 0.90; test-retest reliability = 0.73). In the present study, Cronbach's α for this scale was 0.927.

#### Relationship Assessment Scale (RAS)

The Relationship Assessment Scale (RAS; Hendrick et al., 1998) is a 7-item measure of relationship satisfaction rated on a 5-point Likert scale, with items four and seven reverse-scored; higher scores indicate greater satisfaction with the intimate relationship. The Chinese version used in this study is from Miller (2015b). Prior research using the same Chinese version with Chinese adults in romantic relationships has supported its reliability, reporting an internal consistency of α = 0.86 (Yang et al., 2019). In the present study, Cronbach's α for this scale was 0.751.

#### Attention check items

Two attention-check items were embedded in the questionnaire (items 49 and 57), which instructed participants to “Please choose the option that does not match” and “Please choose the middle option for this question,” respectively. These items were used to identify inattentive or careless respondents and thereby improve data quality. Participants who failed to respond as instructed on these items were considered inattentive and were excluded from the analyses.

### Statistical analysis

This study conducted a Pearson correlation analysis among the two dimensions of adult attachment, perceived partner responsiveness, and intimate relationship satisfaction. To test the mediating role of perceived partner responsiveness in the associations of attachment avoidance and attachment anxiety with relationship satisfaction, a mediation analysis was conducted using Model 4 of the PROCESS macro for SPSS (Hayes, 2018). Because attachment avoidance and attachment anxiety are moderately correlated, the two dimensions were entered into the model simultaneously so that the unique effect of each dimension through perceived responsiveness could be estimated while controlling for the other. Birthplace and relationship duration were included as covariates. For each attachment dimension, path a (the effect of the attachment dimension on perceived responsiveness) and path b (the effect of perceived responsiveness on satisfaction) were estimated, along with the total effect (c), the direct effect (c′), and the indirect effect (a × b), with the other attachment dimension entered as a covariate to isolate each dimension's unique contribution. The indirect effect (a × b) was estimated using bias-corrected bootstrap resampling with 5,000 samples, obtaining 95% confidence intervals; an indirect effect was considered statistically significant when its 95% confidence interval excluded zero. Standardized coefficients (β) are reported.

## Results

### Test for common method bias

Because all data were collected through self-report at a single time point, Harman's single-factor test was performed to examine common method bias (Podsakoff et al., 2003). An exploratory factor analysis entering all items simultaneously, using principal components extraction without rotation, yielded 10 factors with eigenvalues greater than one. The first (largest) factor accounted for 25.73% of the total variance, well below the 40% threshold. These results suggest that common method bias was unlikely to be a serious concern in the present study.

### Descriptive statistics

A total of 559 questionnaires were initially collected. Invalid responses were excluded based on the following criteria: not currently in a relationship or never having been in one (*n* = 131), failed attention checks (*n* = 37), completion time shorter than 64 s (*n* = 24), and straight-line responding (*n* = 9). Some responses met more than one exclusion criterion; therefore, the criterion-specific counts were not mutually exclusive. In total, 155 unique responses were excluded, leaving 404 valid cases for analysis.

The sample included 255 female students (63.1%) and 149 male students (36.9%). In terms of geographical background, 241 participants (59.7%) hailed from urban areas, while 163 (40.3%) were from rural areas. Regarding relationship duration, 41 participants (10.2%) reported being in a relationship for less than 3 months, 84 (20.8%) for 3–6 months, 104 (25.7%) for 6–12 months, 139 (34.4%) for 1–3 years, and 36 (8.9%) for over 3 years.

### Correlation analyses

Pearson correlation analyses were performed among the two dimensions of adult attachment, perceived partner responsiveness, and intimate relationship satisfaction (see Table 1).

**Table 1:** Descriptive statistics and correlations of study variables.

Variable	*M ±SD*	1	2	3	4
1. Attachment avoidance	3.12 ± 0.83	-			
2. Attachment anxiety	3.96 ± 1.07	0.43^*^	-		
3. Perceived responsiveness	5.65 ± 0.84	−0.58^*^	−0.33^*^	-	
4. Relationship satisfaction	3.84 ± 0.62	−0.49^*^	−0.28^*^	0.45^*^	-

*N* = 404. ^*^p < 0.01.

According to the results in Table 1, for the main variables of this study, there is a moderate positive correlation between attachment avoidance and attachment anxiety with statistical significance (*r* = 0.43, *p* < 0.01), indicating a covariant relationship between the level of attachment avoidance and attachment anxiety, indicating that higher attachment avoidance tends to co-occur with higher attachment anxiety.

There is a strong negative correlation between attachment avoidance and perceived partner responsiveness, while there is a moderate negative correlation between attachment anxiety and perceived partner responsiveness. Both are statistically significant (*r* = −0.58, *p* < 0.01; *r* = −0.33, *p* < 0.01), indicating that the higher the level of insecure attachment, the lower the perceived partner responsiveness. There is a moderate negative correlation between attachment avoidance and relationship satisfaction, and a weak negative correlation between attachment anxiety and relationship satisfaction (*r* = −0.49, *p* < 0.01; *r* = −0.28, *p* < 0.01), indicating that the higher the level of insecure attachment, the lower the satisfaction with intimate relationships.

There is a moderate positive correlation between perceived partner responsiveness and intimate relationship satisfaction (*r* = 0.45, *p* < 0.01), indicating that greater perceived partner responsiveness is associated with higher satisfaction.

### The regression analyses

A combined mediation model was estimated in which attachment avoidance and attachment anxiety were entered simultaneously, while controlling for birthplace and relationship duration (see Figure 1). This approach allowed the unique associations of each attachment dimension with perceived partner responsiveness and relationship satisfaction to be estimated while accounting for their shared variance.

**Figure 1:**
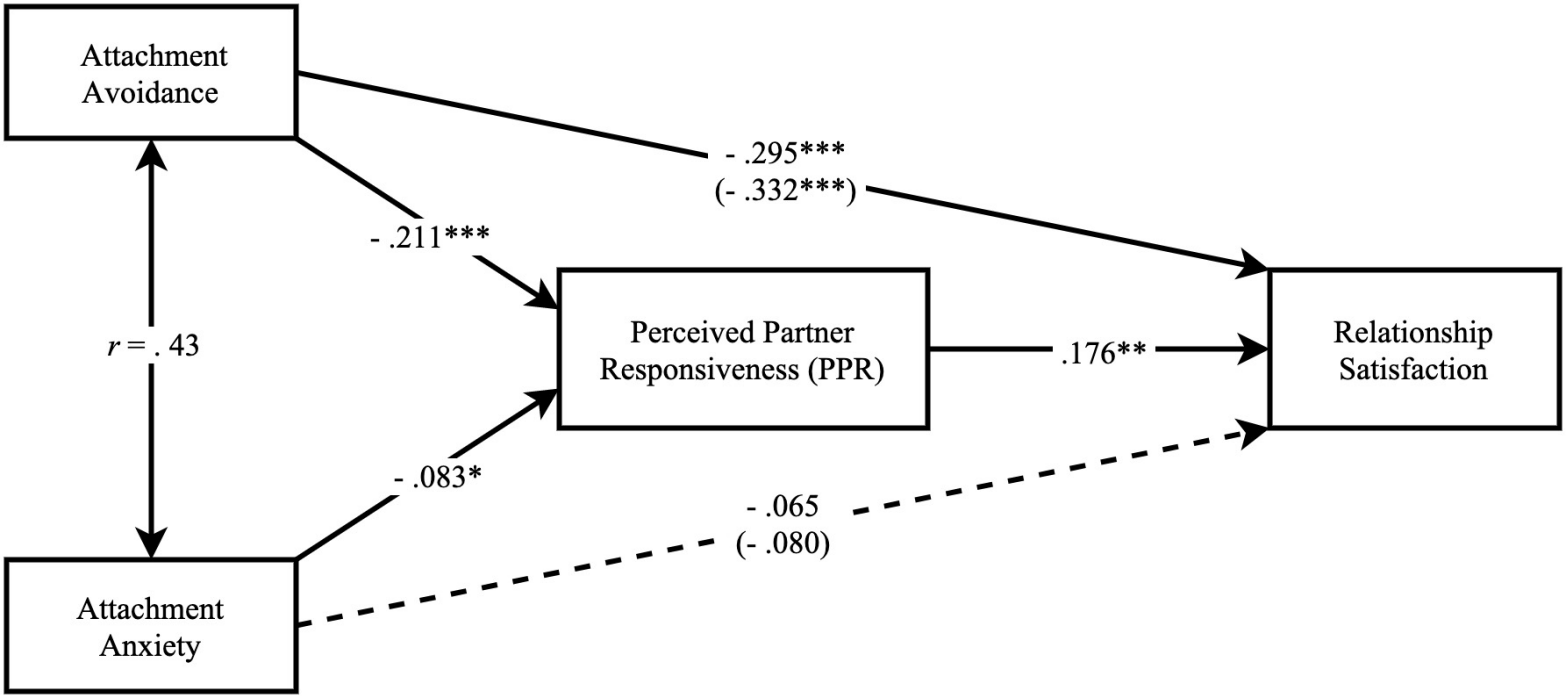
Standardized mediation model of the associations between attachment avoidance, attachment anxiety, and relationship satisfaction via perceived partner responsiveness. Standardized regression coefficients (β) are shown. Coefficients in parentheses represent total effects; unparenthesised coefficients represent direct effects. The double-headed arrow represents the correlation between attachment avoidance and attachment anxiety. Attachment avoidance and attachment anxiety were entered simultaneously, so each path represents the unique association of that dimension while controlling for the other. Birthplace and relationship duration were included as covariates but are not shown. Dashed lines represent non-significant paths. *p < 0.05. **p < 0.01. ***p < 0.001.

For attachment avoidance, the total effect on relationship satisfaction was significant (β = −0.332, *p* < 0.001). Attachment avoidance was significantly and negatively associated with perceived partner responsiveness (β = −0.211, *p* < 0.001), whereas perceived partner responsiveness was positively associated with relationship satisfaction (β = 0.176, *p* = 0.009). The direct effect of attachment avoidance on relationship satisfaction remained significant after accounting for perceived partner responsiveness (β = −0.295, *p* < 0.001).

For attachment anxiety, the total effect on relationship satisfaction was not significant (β = −0.080, *p* = 0.093), nor was the direct effect (β = −0.065, *p* = 0.169). However, attachment anxiety was significantly and negatively associated with perceived partner responsiveness (β = −0.083, *p* < 0.05), whereas perceived partner responsiveness was positively associated with relationship satisfaction (β = 0.176, *p* < 0.01).

### Mediation effect of perceived partner responsiveness

For attachment avoidance, the indirect association with relationship satisfaction through perceived partner responsiveness was statistically significant, as the 95% bias-corrected confidence interval excluded zero (standardized indirect effect = −0.037, 95% CI [−0.084, −0.002]). The direct association between attachment avoidance and relationship satisfaction also remained significant (β = −0.295, *p* < 0.001). Thus, attachment avoidance was associated with relationship satisfaction both directly and indirectly through perceived partner responsiveness.

For attachment anxiety, the indirect association with relationship satisfaction through perceived partner responsiveness was also statistically significant (standardized indirect effect = −0.015, 95% CI [−0.046, −0.001]). However, neither the total association (β = −0.080, *p* = 0.093) nor the direct association (β = −0.065, *p* = 0.169) was statistically significant after controlling for attachment avoidance. This pattern indicates a significant statistical indirect association between attachment anxiety and relationship satisfaction through perceived partner responsiveness.

## Discussion

The present findings showed different patterns for attachment avoidance and attachment anxiety after accounting for the overlap between the two attachment dimensions. Attachment avoidance was significantly associated with relationship satisfaction both directly and indirectly through perceived partner responsiveness. The standardized indirect effect was small in magnitude and accounted for approximately 11.2% of the total association. In contrast, attachment anxiety showed a significant indirect association with relationship satisfaction through perceived partner responsiveness, whereas its unique total and direct associations with relationship satisfaction were not statistically significant after controlling for attachment avoidance. Nevertheless, this does not preclude the presence of a significant indirect association, as a significant total effect is not a prerequisite for detecting an indirect effect (Hayes, 2009; Rucker et al., 2011; Zhao et al., 2010). Total and indirect effects represent distinct statistical quantities, and contemporary mediation approaches therefore recommend testing the indirect effect directly rather than requiring a significant overall association first (Hayes, 2009; Rucker et al., 2011). Therefore, these findings indicate a significant indirect association between attachment anxiety and relationship satisfaction through perceived partner responsiveness, despite the absence of a significant unique total or direct association.

For attachment avoidance, the remaining significant direct association indicates that perceived partner responsiveness accounts for only part of its association with relationship satisfaction. This pattern is consistent with the possibility that other relational or emotional processes may also be involved. Individuals higher in attachment avoidance are often characterized by deactivating strategies, including maintaining emotional distance, suppressing attachment needs, and downplaying attachment-related cues (Simpson and Overall, 2014). They may also be less receptive to or more likely to discount partners' supportive behaviors, which may relate to relationship satisfaction through pathways not fully captured by perceived partner responsiveness.

For attachment anxiety, the significant indirect association through perceived partner responsiveness, together with the absence of significant direct and total associations, suggests that perceptions of partner responsiveness may be particularly relevant to the statistical association between anxiety and relationship satisfaction. This pattern may be understood in light of the hyperactivating strategies characteristic of attachment anxiety. Individuals higher in attachment anxiety tend to seek reassurance, remain highly alert to signs of rejection or abandonment, and hold strong expectations regarding their partner's responsiveness (Mikulincer and Shaver, 2007; Simpson and Overall, 2014). Perceived partner responsiveness directly reflects whether individuals feel sufficiently validated and supported by their partners, which closely corresponds to the relational needs of anxiously attached individuals and may, in turn, be associated with their relationship satisfaction.

By focusing on young adulthood as a distinctive developmental stage, the present study extends existing attachment research and highlights directions for future investigation. Developing romantic relationships and establishing intimacy are central developmental tasks during this period (Erikson, 1950; Xia et al., 2018), while romantic partners increasingly become important sources of emotional support and attachment, and relationship representations may still be developing in response to ongoing relational experiences (Suh and Fabricius, 2020). In this context, perceived partner responsiveness may represent an especially meaningful relationship-specific appraisal because it reflects whether young adults feel understood, validated, and cared for by a partner who is becoming an increasingly important attachment figure. The significant indirect association observed for attachment anxiety may be particularly relevant in this regard. Individuals higher in attachment anxiety have stronger needs for reassurance and acceptance, and perceived partner responsiveness directly captures whether these needs are experienced as being met within the current relationship. Thus, among young adults, lower perceived responsiveness may be especially a vital path through which attachment anxiety is associated with lower evaluations of relationship quality. However, because the present study did not compare different age groups, future research is needed to determine whether this indirect association is stronger during young adulthood than at later developmental stages.

A positive correlation between attachment avoidance and attachment anxiety was observed in the present sample (*r* = 0.43). Rather than indicating a measurement problem, this pattern is consistent with a large body of research showing that the two attachment dimensions are typically modestly to moderately positively correlated (e.g., Yang et al., 2019). Conceptually, this co-occurrence is plausible because individuals with a history of inconsistent or unresponsive caregiving may develop elevated levels of both dimensions, and the two forms of insecurity share an underlying lack of felt security (Mikulincer and Shaver, 2007). The internal consistency of both ECR subscales in the present sample was high (α = 0.84 for avoidance and 0.91 for anxiety), indicating good reliability. Nonetheless, because the two dimensions were correlated, we modeled them simultaneously so that the unique association of each could be estimated while controlling for the other; this analytic choice directly addresses their shared variance. Additionally, the possibility that avoidance and anxiety are more strongly linked in young adult or Chinese samples remains an open question that future research could examine.

Furthermore, the interpretation of the indirect associations should take into account the cross-sectional nature of the present study. Because all variables were assessed at a single time point, the current design cannot capture how perceived partner responsiveness and relationship satisfaction may develop or influence one another over time. Longitudinal research suggests that perceived responsiveness may be linked to changes in relationship functioning across repeated interactions (Canevello and Crocker, 2010; Selcuk et al., 2017). Thus, although the indirect association observed for attachment avoidance was modest in magnitude, and the indirect association for attachment anxiety was also small, perceived partner responsiveness may still represent a relational process worthy of further longitudinal and intervention-based investigation.

These findings can also be understood through an integration of social exchange theory and cognitive evaluation theory. From a social exchange standpoint, relationship satisfaction reflects individuals' appraisal of whether the emotional support, care, and understanding they receive correspond to what they invest (Thibaut and Kelley, 1959; Miller, 2015a), and perceived partner responsiveness can capture this subjective sense of relational return. Cognitive-evaluation processes help explain why individuals with different attachment orientations appraise the same partner behaviors differently: those higher in avoidance may discount or dismiss caring behaviors, whereas those higher in anxiety may be more sensitive to signs of insufficient responsiveness and interpret ambiguous responses negatively. Together, these perspectives suggest that attachment orientations shape how partner behavior is perceived and translated into a sense of relational return, which in turn is associated with relationship satisfaction.

### Practical implications

The present findings offer tentative implications for relationship education and counseling among college students, although they should be read in light of the study's cross-sectional and correlational nature. Because perceived partner responsiveness was associated with relationship satisfaction across both attachment dimensions, interventions that strengthen individuals' capacity to notice, accurately interpret, and take in their partner's supportive behaviors may be broadly useful for those with insecure attachment, rather than being specific to any single profile.

Within this general aim, some tailoring may be worth exploring. To the extent that avoidance and anxiety are linked to different interpretive tendencies, downplaying or dismissing supportive cues in the former case, and heightened sensitivity to signs of insufficient responsiveness in the latter, counseling could, where appropriate, attend to these differing tendencies. Such distinctions, however, should be treated as points of emphasis within a common focus on responsiveness rather than as separate intervention tracks, particularly because avoidance and anxiety were moderately correlated and frequently co-occur within the same individual.

More broadly, because perceived partner responsiveness is a relational, rather than purely individual, process, dyadic approaches that improve mutual understanding and communication may help increase perceived responsiveness for both partners. University counseling services could integrate attachment-informed and cognitive-behavioral approaches to help students develop more balanced expectations and interpretations of their partners' behavior.

### Limitations

First, the cross-sectional design limits causal interpretation of the present findings. Although significant indirect effects through perceived partner responsiveness were observed, these effects should be interpreted as statistical associations rather than evidence of a causal mediating process. Because attachment, perceived partner responsiveness, and relationship satisfaction were measured at the same time point, the temporal ordering among these variables cannot be established. Longitudinal or experimental research is therefore needed to test whether insecure attachment prospectively predicts changes in perceived partner responsiveness and whether such changes are subsequently associated with relationship satisfaction.

In addition, self-report methods are susceptible to social expectations and cognitive biases. Although Harman's single-factor test indicated that common method bias was unlikely to be severe, this technique is relatively insensitive. In future research, data from multi-source or multi-wave designs should be integrated.

In terms of sample size, this study has an imbalanced gender ratio (with a majority of females) and is limited to Chinese university students, which limits the generalizability of the conclusions. Future research should expand the diversity of the sample (such as age, cultural background, balanced gender, etc.). In addition, this study only controlled for demographic variables and did not consider other possible influencing factors such as social support networks, frequency of relationship conflicts, etc. These variables can be included in future studies to explore other moderating or mediating variables. Moreover, the present sample consisted largely of young adults in relatively early-stage dating relationships. The findings may therefore not generalize to individuals in committed, long-term, or marital relationships, in which attachment dynamics and the role of perceived partner responsiveness may operate differently. Future research should examine these processes across different relationship stages and levels of commitment.

Last but not least, although the internal consistency of the ECR subscales was satisfactory, attachment avoidance and anxiety were positively correlated in this sample. As noted above, this is consistent with prior research; nonetheless, the degree of overlap between the two dimensions should be kept in mind when interpreting their unique associations, and future work could further examine the discriminant validity of the two subscales in emerging-adult and Chinese samples.

## Conclusion

Guided by attachment theory, the present study examined the associations of attachment avoidance and attachment anxiety with relationship satisfaction among Chinese college students in young adulthood, and whether perceived partner responsiveness helped account for these associations. By entering both attachment dimensions simultaneously, the study was able to distinguish their unique associations while accounting for the variance they share. Focusing on young adults also extends existing research to a developmental period in which establishing and maintaining intimate relationships is particularly salient.

The findings revealed different patterns for the two attachment dimensions. Attachment avoidance was negatively associated with relationship satisfaction both directly and indirectly through lower perceived partner responsiveness. In contrast, after controlling for attachment avoidance, attachment anxiety was not uniquely associated with relationship satisfaction at the direct or total level, but showed a small significant indirect association through perceived partner responsiveness. These findings suggest that perceived partner responsiveness may play different roles in the associations of attachment avoidance and attachment anxiety with relationship satisfaction among young adults. More broadly, perceived partner responsiveness may represent an important relationship-specific appraisal in understanding how attachment insecurity is associated with evaluations of romantic relationship quality during young adulthood. Future research could compare individuals across different developmental stages to examine whether the role of perceived partner responsiveness is particularly salient during young adulthood. Given the cross-sectional design, these findings should be interpreted as statistical associations rather than evidence of causal mediation.

## Data Availability

The datasets presented in this study can be found in online repositories. The names of the repository/repositories and accession number(s) can be found below: https://osf.io/c4fpq?view_only=22fc3b31a9474879853c7ad42666d318.
